# Involvement of *Gonolabis distincta* in the Control of Root Maggots in Garlic Fields

**DOI:** 10.3390/life15081192

**Published:** 2025-07-27

**Authors:** Caihong Tian, Junpeng Li, Yan Zhang, Junyi Zhang, Xinju Gao, Xinming Yin, Lirong Yang, Hongqiang Feng

**Affiliations:** 1 Henan Key Laboratory of Agricultural Pest Monitoring and Control, Key Laboratory of Integrated Crop Pests Management on Crops in Southern Region of North China, Ministry of Agriculture and Rural Affairs, Agricultural Microbiology Innovation Center of Henan Province, No. 0 Entomological Radar Field Scientific Observation and Research Station of Henan Province, Institute of Plant Protection, Henan Academy of Agricultural Sciences, Zhengzhou 450002, China; caihongtian@hnagri.org.cn (C.T.); lijunpeng0815@163.com (J.L.); zjy2024153708@163.com (J.Z.); gaoxj19@126.com (X.G.); 2 Institute of Agricultural Information Technology, Henan Academy of Agricultural Sciences, Zhengzhou 450002, China; yzhang1203@hnagri.org.cn; 3 Department of Biological Engineering, Henan University of Animal Husbandry and Economy, Zhengzhou 450002, China; xinmingyin@hotmail.com

**Keywords:** garlic pests, root maggot, DNA barcoding, *Gonolabis distincta*, predation ability

## Abstract

Garlic root maggots are the main pest of garlic in Qi County, Henan Province, China, which has become an important factor restricting the development of the garlic industry. Earwigs play an important role in controlling root maggots because of their similar ecological niches. In this study, through DNA barcoding and morphological identification, the following root maggots and main earwigs species from Qi County were quickly identified: *Delia platura* (Meigen), *Bradysia odoriphaga* Yang et Zhang, *Delia antiqua* (Meigen), *Muscina angustifrons* (Loew), *Lucilia sericata* (Meigan), and the main species of earwigs was *Gonolabis marginalis* (Dohrn). *D. platura* was the dominant species and accounted for 98% among all garlic root maggots. The predation ability for each stage of *G. distincta* on the larvae and pupae of *D. platura* showed that *G. distincta* at different developmental stages preyed on both the the larvae and the entire pupae of *D. platura*. Among them, female adults had the strongest predation ability and the largest daily predation on first instar larvae of gray *D. platura* (71.25 ± 0.66). First instar nymphs of *G. distincta* also had a certain predation ability with the daily predation of first instar larvae being (1.85 ± 0.13). The predation ability of *G. distincta* at different instars on the larvae of the same instar of *D. platura* increased with the increasing of the instar. For the first to second instar larvae of *D. platura*, the female adult of *G. distincta* had the strongest predation ability, followed by the male adult of *G. distincta*, and then the fifth instar nymph of *G. distincta*. There was no significant difference in the predation ability between the male and female adults of *G. distincta*, but the adults’ predation capacities were significantly higher than that of the fifth instar nymph of *G. distincta*. The capacity of the fifth instar nymph of *G. distincta* was significantly higher than the fourth instar nymph of *G. distincta*, the fourth instar nymph of *G. distincta* was significantly higher than the first to third instar nymphs, and there was no significant difference in the predation amount among the first to third instar nymphs. The predation selection experiment indicated that the fifth instar nymphs and the male and female adults of *G. distincta* showed a positive preference for the first to third instar larvae of *D. platura* and a negative preference for the pupae of *D. platura*. Our study provided a preliminary scientific basis for green prevention and control of garlic root maggot.

## 1. Introduction

Garlic (*Allium sativum* L.) is a lily family herb and a spicy vegetable. It has been cultivated for more than 2000 years in China. It is loved by consumers as a traditional medicine for food and the treatment of various diseases. [1]. With the improvement of people’s living standards, health is being paid more and more attention. As a health food, garlic and its products are becoming more and more popular among people all over the world. Garlic is not only a condiment but also can sterilize, has the effect of lowering blood fat, fighting cancer, and is a traditional agricultural product in China. China is one of the largest producers in the world, also a consumer and exporter of garlic, accounting for more than half of the worlds total exports. With the increase in garlic demand domestic and overseas, the planting area and output of garlic in China are increasing continuously. In 2021, Chinese garlic planting area was 9 × 10^9^ m^2^. Chinese garlic output was 21.625 million tons.

Qi County, Henan Province is rich in agricultural resources, known as the “granary of the Central Plains”, and is famous locally and abroad as “the hometown of Garlic in China”. The planting area has remained stable at over 700,000 mu for 15 consecutive years, with an annual output exceeding 900,000 tons. The planting scale and total output have firmly ranked first among counties nationwide, forming a complete industrial chain system covering planting, processing, cold chain logistics, and trade. In 2024, the direct export volume reached USD 515 million, and the products were sold far to more than 50 countries including the European Union and Southeast Asia. It had been rated as a national-level export garlic quality and safety demonstration area. In 2023, the brand value was evaluated at CNY 5.612 billion, becoming an important reference for global garlic pricing [2]. It was one of the three core producing areas for the development of the garlic industry in China, and its planting area and annual output ranked first in counties in China.

Qi County is a national demonstration area for exporting garlic quality and safety and an advantage area of agricultural products with Chinese characteristics. It has built a modern agricultural industrial park in Henan Province with garlic characteristics and the only provincial quality supervision and inspection center for garlic and garlic products in Henan Province. “Qi County garlic” is the “The Ministry of Agriculture and Rural Affairs agricultural products geographical indication registration products”, “Chinese 100 geographical indications” products, the national “geographical indication certification trademark”. It is the pillar industry of the county’s agricultural economy and farmer’s income. Therefore, the green and efficient planting of garlic in Qi County has become a major measure to promote the high-quality development of agriculture in Qi County and fully implement the rural revitalization strategy.

However, garlic in Qi County is facing increasing soil pests like the root maggot year by year [3]. Root maggots mainly harm the stem of garlic seedlings, resulting in yellow leaves, small plants, fragility, rot, heaviness, and even death. The annual rate of plants affected can reach 20% to 50%, with a mortality rate of 10% to 20% [4]. In order to prevent and control garlic root maggots, farmers root pour, resulting in excessive pesticide residues, which seriously affects the quality of garlic maggots. Unclear pest species and lagging prevention and control have become important factors restricting the development of the garlic industry, and a set of safe and effective prevention and control measures are urgently needed in production. Traditional Diptera identification is based on morphology, but for similar species like root maggots, morphological identification has great limitations, therefore an accurate, rapid identification method is needed to quickly identify garlic root maggots to develop precise control strategy.

Earwigs are representatives of insects in the order Dermaptera widely distributed in the Huang-Huai-Hai fields. Most species of earwigs are predatory and prefer to catch live insects. Indoor experiments have also found that these insects like to feed on young live insects [5]. Moreover, the growth cycles of their nymphs and adults are long, and they have strong environmental adaptability. What are the species of earwigs in garlic fields? How effective are they in controlling garlic maggots? Until now, there is no reported case yet.

There are over 1000 species of maggots. In the study, we employed DNA barcode identification as a crucial step to distinguish between various species of root maggots, enhancing the precision and reliability of our findings [6,7]. Garlic root maggots collected from garlic fields and earwigs trapped in the same garlic field were identified using DNA barcoding technology and morphological observation. The predatory ability of the main species of earwig against the dominant garlic root maggots was determined under laboratory conditions.

## 2. Materials and Methods

### 2.1. Insects

From 1 December 2023 to 9 June 2025, 80 survey sites in Qi County were randomly selected in the garlic field of Qi County by satellite remote sensing, and the larvae, pupae, and adults of root maggots were collected for experiments. Earwigs were collected from garlic field, Caotun village, Pei Cundian Town, Kaifeng County, Henan Province. The adults were lured by traps and bait [8] with temperature (27.2 ± 0.5) °C, relative humidity (80 ± 5)%, and photoperiod L/D = 16 h/8 h. The dominant earwig predator was starved for 24 h prior to the experiment.

### 2.2. Morphological Identification

Different developmental stages of garlic root maggots and earwigs were photographed using the super-depth of field 3D microscope (Keenz VHX-500F, Keenz Limited, Tokyo, Japan) to observe and record the dominant root maggot species’ main external morphological characteristics of larvae, pupae, and adults.

### 2.3. DNA Barcode Identification

#### 2.3.1. Extraction of the Sample for Genomic DNA

Garlic root maggots were collected from 80 randomly selected sample points in Qi County using satellite remote sensing. Samples of larvae, pupae, or adults were washed with distilled water and transferred into a 1.5 mL centrifuge tube. The DNA extraction was performed using Tissue DNA Kit (50) Omega D3396-01. An appropriate amount of liquid nitrogen was added into the samples and the samples were ground. Following the kit’s instructions, 350 μL of CTL buffer and 25 μL of proteinase K solution were mixed in the centrifuge tube and incubated at 60 °C for 30 min until the entire sample was dissolved. Subsequently, 350 μL of chloroform/isoamyl alcohol (24:1) was added and thoroughly mixed before centrifuging at 10,000× *g* for 2 min at room temperature. The upper aqueous phase was carefully transferred to a clean 1.5 mL microcentrifuge tube, taking care to avoid the milky white interfaces that contain contaminants and inhibitors. One volume of CBL buffer and 2 μL of RNase were added, followed by vortexing for 15 s. The mixture was then incubated at 70 °C for 10 min, after which 100% ethanol was added and vortexed at maximum speed for 15 s. Carefully, 750 μL of the cleared lysate, including any potential precipitate, was aspirated into the HiBind^®^ DNA mini column. The HiBind^®^ DNA mini column was then inserted into a 2 mL collection tube and centrifuged for 1 min, after which the filtrate was discarded. The collection tube was reused, and this step was repeated until all remaining samples were transferred to the HiBind^®^ DNA mini column. The HiBind^®^ DNA mini column was transferred to a new 2 mL collection tube, and 250 μL of HB buffer was added. The mixture was centrifuged at 10,000× *g* for 30 s, the filtrate was discarded, and the collection tube was reused. This step was repeated twice. Next, 350 μL of DNA wash buffer was added, and the mixture was centrifuged at 10,000× *g* for 1 min. The filtrate was discarded, and the collection tube was reused. This step was repeated four times. The empty HiBind^®^ DNA mini column was centrifuged at maximum speed for 2 min to dry the column matrix. The HiBind^®^ DNA mini column was then transferred to a clean 1.5 mL microcentrifuge tube, and 20 μL of sterile, deionized water, heated to 70 °C, was directly added to the center of the column membrane. The tube was left at room temperature for 2 min and then centrifuged at 10,000× *g* for 1 min. This step was repeated twice. Finally, the samples were clearly marked and stored at −20 °C.

#### 2.3.2. Polymerase Chain Reaction (PCR) and Sequence Determination

The COI gene fragments were procured using PCR amplification. The primer sequences used were LCO1490: 5′-GGTCAACAAATCATAAAGATATTGG-3′ and HCO2198: 5′-TAAACTTCAGGGTGACCAAAAAATCA-3′ [9]. The PCR mixture totaled 25 μL, including ddH2O 18.3 μL, 10× Ex Taq buffer (with Mg^2+^) 2.5 μL, dNTPMix 2 μL, 1 μL of DNA template, 0.5 μL of each primer (20 μM), Taq DNA Polymerase (5 U/μL), and 0.2 μL. The PCR conditions were as follows: initial denaturation at 95 °C for 3 min; followed by 34 cycles of 94 °C for 30 s, 95 °C for 30 s, and 55 °C for 30 s; with a final extension at 72 °C for 5 min and storage at 12 °C. The resulting PCR products, after amplification with the primers, were purified and sequenced by Bioengineering (Shanghai) Co., Ltd., Shanghai, China.

#### 2.3.3. Sequence Analysis

Upon obtaining the sequencing results, the sequencing peak map was examined to ascertain the accuracy of the sequencing process. DNASTARv11.1 software was utilized for sequence assembly, during which the primer sequence was excised [10], and a COI gene sequence fragment of approximately 700 bp was acquired and saved in FASTA format. Sequence alignment was performed using Standard Nucleotide BLAST accessed on 10 June 2024 (https://blast.ncbi.nlm.nih.gov/Blast.cgi?PROGRAM=blastn&PAGE_TYPE=BlastSearch&BLAST_SPEC=&LINK_LOC=blasttab&LAST_PAGE=blastn). Sequence divergences were evaluated using the Kimura two-parameter (K2P) distance model. Bootstrapping was executed in MEGA12 with 1000 replications.

### 2.4. Predation Behavior of Domiant Earwig Species on Dominant Root Maggot Species

The dominant species of garlic root maggots was identified according to Section 2.3.1. under laboratory conditions. The, 1st to 5th instar nymphs emerged on the same day, and male and female adults before mating were starved in a circular breeding box (upper diameter = 15 cm, lower diameter = 17 cm, height diameter = 9 cm; the same below) for 24 h. During the starvation period, sterilized distilled water was used to moisten cotton balls, which were then added to replenish the water for the test worms. Simultaneously, *D. platura* larvae were provided with garlic bulb to ensure they had sufficient food. Based on the results of the pilot experiment, 60 first to third instar *D. platura* larvae were placed into each box. Each treatment set included a control. The predator control box contained only distilled water and a cotton ball, while the prey control box had only first to third instar *D. platura* larvae with artificial feed. Each was repeated 20 times, and the number of naturally deceased insects was recorded. Using a video microscope (3DM-micro, HD202WF, Shenzhen Co., Ltd., Shenzhen, China), the number of surviving *D. platura* larvae under different treatments was recorded after 24 h. Each treatment included only the corresponding density of *D. platura* larvae as a negative control, and survival was assessed after 24 h. All experimental conditions were kept consistent throughout.

#### 2.4.1. Determination of Predation Preferences of Larvae and Pupae by Nymph and Adults of Dominant Earwig Species

Robust 5th instar nymphs, male and female adults of both prey and predators, were selected and starved overnight in the previously described box for 24 h. Based on the results of a pilot experiment, 10 larvae (including a bulb of garlic), pupae, male and female adults (including a honeypot filled with adult nutrient solution), were combined in a box (length, width, and height = 250 mm, 180 mm, 100 mm). Then, one individual was placed into the box with hungry predators. The housing was in an artificial climate box with a temperature of (27.2 ± 0.5) °C, a photoperiod of L/D = 16 h/8 h, and relative humidity of (80 ± 5)%. Experiments were conducted from 8:00 to 12:00, with 20 repetitions per treatment. The control treatment was the same as that described in Section 2.4.

#### 2.4.2. Preference of Different States of Dominant Earwig Species on Dominant Root Maggot Species

The predation preference was determined using the preference index value Ci. The formula is Ci = (Qi − Fi)/(Qi + Fi). In this formula, Ci represents the predator-to-prey preference index, Qi indicates the proportion of predators feeding on the prey of ith, and Fi represents the proportion of the ith prey in the environment. Ni represents the number of ith-species prey in the environment, and Nai represents the number of ith prey for predators. Then, Fi = Ni/∑Ni, and Qi = Nai/∑Nai. If Ci ≥ 0, this indicates that predators have a positive preference for the ith prey, while the Ci value being ≤−1 indicates that predators have a negative preference for the ith prey.

### 2.5. Data Analysis

The original data were processed by using comma-separated values (CSVs), and then variance analysis (ANOVA) and paired comparison methods were used to test the significant differences in the average predation under different treatment conditions, using R-4.4.2 software to complete. The processed group data were presented in the form of mean ± standard error (SE). Multiple comparisons were performed using Duncan’s new multiple range test.

## 3. Results and Analyses

### 3.1. Observation Point Setting and Garlic Root Maggot Sampling

**Figure 1:**
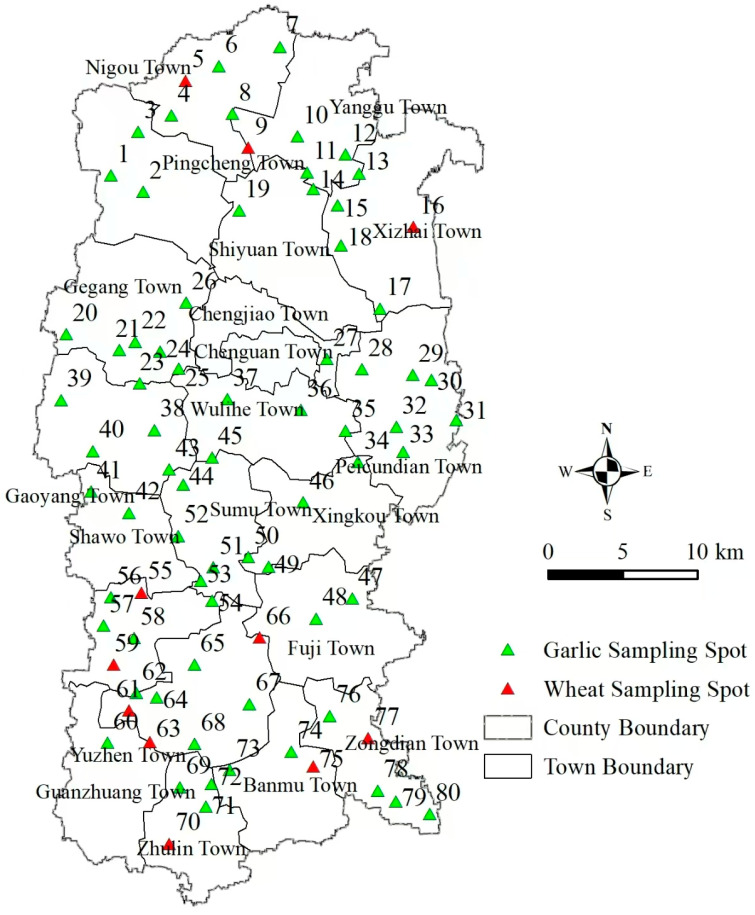
Satellite remote sensing identified 80 survey sites across 21 townships in Qi County, Henan Province, China. The green triangular symbols represent garlic sampling spots, while the red triangular symbols represent wheat sampling spots.

Through satellite positioning, at the 80 sampling points selected in 21 towns in the county (excluding villages roads and ditches) (Figure 1), about 2000 root maggots were brought back to the laboratory each time. After sorting in the laboratory and taking photos under the microscope, the insect samples were reared in an artificial climate chamber.

### 3.2. Identification of Root Maggots and Earwigs Collected from Garlic Field

#### 3.2.1. Species Compositions, Morphological Descriptions, and Life Cycle Observations

From 1 December 2023 to 9 June 2025, root maggots collected from 80 sample sites in 21 townships of Qi County, Henan Province, were observed by microscope. Genomic DNA was extracted as a template for Polymerase Chain Reaction (PCR). Mitochondrial COI (Cytochrome Oxidase I) of the target sample was obtained by PCR amplification. The DNA sequences were obtained by PCR sequencing. The results of comparing the sequence with GenBank and BOLD databases showed that the root maggots collected in garlic fields were flies and mosquitoes. Species of root maggots were named as *Delia platura* (Meigen), *Bradysia odoriphaga* Yang et Zhang, *D. antiqua* (Meigen), *Muscina angustifrons* (Loew), and *Lucilia sericata* (Meigan). The main species of earwigs is *Gonolabis marginalis* (Dohrn) (Table 1).

**Figure 2:**
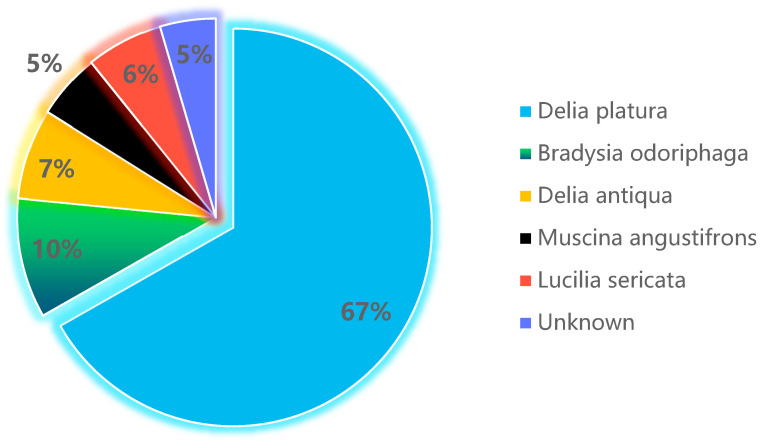
Occurrence types and percentages of garlic root maggots in Qixian County.

The dominant species was the gray ground species *D. platura*, which possessed the highest proportion, accounting for 67% among the total county sampling sites. The second was *B. odoriphaga*, possessing 10% of the whole county sampling sites. The others were *D. antiqua*, *M. angustifrons*, and *L. sericata*, which possessed 7%, 5%, and 6%, respectively, and 5% of the unknown species (Figure 2).

*D. platura* possessed the highest proportion, accounting for 67% among the total county sampling sites (blue color). The second was *B. odoriphaga*, possessing 10% of the whole county sampling sites (green color). The others were *D. antiqua* (yellow color), *M. angustifrons* (black color), and *L. sericata* (purplish blue), which possessed 7%, 5%, and 6%, respectively, and 5% of the unknown species.

**Figure 3:**
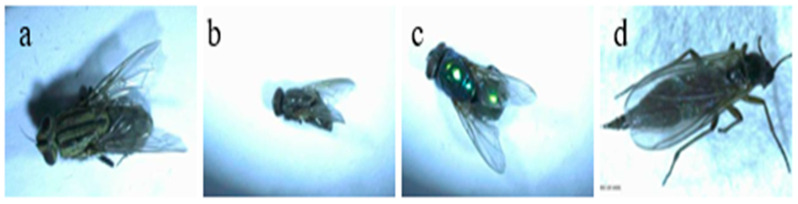
Non-dominant species in garlic fields: (**a**) *Muscina angustifrons*; (**b**) *Delia antiqua*; (**c**) *Lucilia sericata*; and (**d**) *Bradysia odoriphaga*.

There were also other root maggots in the garlic field in Qi County. They were *D. antiqua* (Figure 3a), *M. angustifrons* (Figure 3b), *L. sericata* (Figure 3c), and *B. odoriphaga* (Figure 3d), which possessed 7%, 5%, 5%, and 6%, respectively. There was still 5% of the unknown species that needed to be determined.

#### 3.2.2. Subculture Rearing of *D. platura*

**Figure 4:**
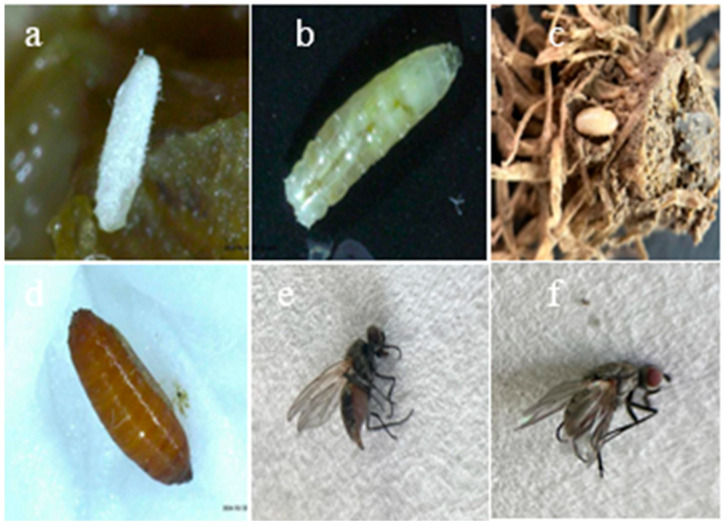
The life cycle of *Delia platura*: (**a**) egg; (**b**) larvae; (**c**) prepupa; (**d**) pupa; (**e**) female adult; and (**f**) male adult.

The dominant species, *D. platura*, was collected from the garlic field and reared with garlic bulb in the artificial climate box under the temperature of (27.2 ± 0.5) °C, photoperiod of L/D = 16 h/8 h, and relative humidity of (80 ± 5)%. Through multi-generational feeding, we observed that the eggs were oval or long oval, milky white to pale yellow, with a smooth surface (Figure 4a). The eggs hatched into larvae after three days. Female adults oviposited eggs at the junction of the garlic head and stem. The mature eggs measured 1.0 ± 0.35 mm in length, were oblong, and slightly curved. Mature larvae had a body length of 4–6 mm, were light white to light yellow, and had a degraded head. They possessed only one pair of black hooks (Figure 4b). The front end of the insect body was thin, while the back end was thick, and there were seven pairs of fleshy protrusions at the tail end. The pupae were 4 to 5 mm in length, reddish brown or yellowish brown, oval, slightly flattened at the front end, and rounded at the back end with several protrusions (Figure 4c,d).

The adult body length was 4–6 mm. The male was dark brown, with compound eyes nearly touching each other. The antennae were black and awn-shaped. There was a black longitudinal stripe in the center of the abdomen’s back and a black transverse stripe in each abdominal internode, with black feet. For male adults, the inner side of the hind tibia had rows of dense, end-curved, equal-length hairs, and three long hairs on the outer side. However, the female did not have these features. The female’s body color was slightly lighter, yellow or yellowish brown, and the distance between the abdomen and the eye was wide, approximately 1/3 of the head width. There were three brown longitudinal stripes on the back, and the central longitudinal stripes on the abdomen were not obvious. There was only one seta on the anterolateral side of the middle tibia of the middle foot, and the abdomen was cylindrical and tapered to a rounded tip at the end. Four to five segments of the tergum were visible, and the remaining segments formed the terminalia. In female flies, the terminalia typically formed an ovipositor that extended during oviposition. The characteristics of the male external genitalia were important bases for the identification of fly species (Figure 4e,f).

### 3.3. Predation Behavior, Predation Capacity, and Predation Preference

#### 3.3.1. Predation Behavior of *G. marginalis*

**Figure 5:**
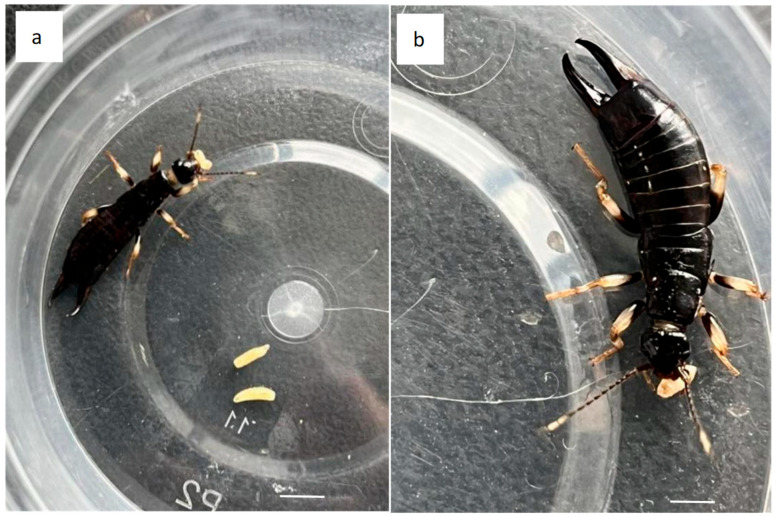
The male and female adults of *Gonolabis distincta* prey on the third instar larvae of *Delia platura.* (**a**) Male adult of *G. distincta* preying on the third instar larvae of *D. platura*; (**b**) female adult of *G. distincta* preying on the third instar larvae of *D. platura*; bar = 1 mm.

Under laboratory conditions, nymphs from the first to fifth instar larvae and both male and female adults of *G. Marginalis* were observed to prey on the first to third instar larvae and pupae of *D. platura*, demonstrating a strong capacity for control. During the predation process, they exhibited a four-stage process: rapid crawling, followed by an up-and-down jittering search with their antennae, then testing with their mouthparts and feet, and finally securing the prey with a tail clamp before biting. After consuming the entire larval body, they would proceed to seek out additional *D. platura* larvae. For third instar larvae, they would first avoid one side, then gently probe before quickly securing a tail clamp. They would avoid the hard head of the *D. platura* larvae and use their chewing apparatus to first tear the prey’s skin, then chew the body, and subsequently search for more *D. platura* larvae. In observing the predation behavior, no significant difference was noted between male and female adults (Figure 5).

During the experiment, it was observed that when satiated, male and female adults would consume only a small portion of the prey after biting through the body wall, until the larvae ceased to struggle. They would then abandon the prey and proceed to search for additional prey to continue their hunt.

#### 3.3.2. Predation Ability of *G*. *distincta* Against Larvae and Pupae of *D*. *platura*

Our findings indicated that *G. distincta* at all developmental stages can feed on various stages of larvae and pupae of the *D. platura*. The predation rate on larvae at different developmental stages of *D. platura* decreased as the instar increased. Female adults exhibited the strongest predatory ability, with a maximum daily predation of (71.25 ± 0.66) individuals, which was 38.52 times greater than that of the first instar larvae. Male adults followed with a daily predation of (63.85 ± 0.41). The first instar larvae of *G. distincta* also possessed predatory capabilities, with an average daily predation of (1.85 ± 0.13). Our results suggested that *G. distincta* can effectively control *D. platura* at various developmental stages, particularly the younger larvae. The predation rate on *D. platura* increased with the instar stage of the larvae. There was no significant difference in the predation rate among the first to third instar nymphs, with daily averages ranging between 1.85, 2.05, and 2.45, respectively. The predation preference of *G. distincta* at various developmental stages was significantly higher for the younger larvae (first–second instars) than for the older larvae and pupae (difference in lowercase letters in the same row). For example, the amount the first instar larvae was preyed upon by female adults (71.25) is 2.27 times that of pupae (31.40), indicating that the hardening of the prey’s cuticle or morphological changes may reduce the risk of predation (Table 2).

The predation amount of female adults was significantly higher than that of males at all prey stages (for example, for predating third instar larvae: female 57.70 ± 0.54 vs. male 42.75 ± 0.41). The fourth instar nymph showed a significant increase in predation ability (the predation amount was 15–20 times higher than that of the third instar larvae), suggesting that this stage was a critical developmental threshold for *G. distincta* from inefficient predation to efficient predation. However, when comparing with younger larvae of *D. platura* with the fourth instar of *D. platura*, there was no significant difference between the male and female adults (Table 2).

#### 3.3.3. Predatory Preference of Fifth Instar Nymphs and Male and Female Adults of *G. distincta* Against Different Developmental Stages of *D. platura*

For the first to second instar larvae of *D. platura*, the whole body could be eaten. But for elderly larvae like the third instar larvae of *D. platura*, *G. distincta* would absorb the body fluids to leave the epidermis empty shell. In the experiment, it was observed that the fifth instar nymphs of *G. distincta* were sometimes surrounded by *D. platura* larvae, but they were quickly frightened by the shaking of the tail clip and then released of niff. After multiple repeated experiments, the fifth instar nymphs were most likely to prey on the first and second instar larvae of *D. platura*, with the predation amount being 27.75. Among the preys composed of different developmental stages, female adults showed positive preference to all the developmental stages of *D. platura*. However, the fifth instar nymphs and male adults of *G. distincta* showed negative preference to pupae of *D. platura* with *Ci* = −0.11 and *Ci* = −0.14, respectively. It was significantly higher than other prey of *D. platura* (*p* < 0.05) (Table 3).

Our findings revealed a distinct predation preference among fifth instar nymph, female and male adults of *G. distincta* for various developmental stages of *D. platura*. Notably, all stages of *G. distincta* showed the highest predation preference for third instar larvae of *D. platura*, as indicated by the highest daily predatory number (PN) and preference index (Ci). The predation preference decreased sequentially from third instar larvae to pupae, with the lowest PN and Ci recorded for pupae.

Furthermore, statistical analysis highlighted significant variations in predation preference among different developmental stages of *G. distincta* when targeting the same developmental stage of *D. platura*. For example, third-instar larvae of *G. distincta* demonstrated a significantly higher PN and Ci for third-instar larvae of *D. platura* compared to other developmental stages of *G. distincta*. Similarly, significant differences were observed among different developmental stages of *D. platura* when preyed upon by the same stage of *G. distincta*. These results indicate that the predation preference of *G. distincta* was influenced by both the developmental stage of the predator and the prey.

## 4. Discussion

An accurate and rapid method for identifying pests is essential for timely control measures. The first method is morphological identification. However, the garlic root maggot species complex was often mixed occurrence, and the high similarity between larvae and eggs made identification difficult. For similar species, especially larvae and eggs, there were great limitations in morphological identification, so it is necessary to find accurate and rapid identification methods for garlic root maggots. Therefore, DNA barcoding technology can be used to quickly and accurately identify the species of pests, which not only shortens the identification time but also improves the accuracy of identification. At present, DNA barcoding technology has played an important role in the identification of insect pests in China, such as Ma et al. (2025) [11]. They used this technology to identify the harmful pomegranate mite *Tenuipalpus hornotinus* [12] and to identify the peach aphid wasp [13]. In this study, species of garlic root maggots were identified using DNA barcoding technology, which aligned with the results of morphological identification. This provided a technical reference for pest prevention and treatment in China and helped to mitigate the damage caused by root maggots to garlic. By integrating traditional morphological methods with DNA barcoding technology, a more precise judgment would greatly facilitate the rapid identification and control of pests in the future.

Furthermore, the application of DNA barcoding technology in this study underscored its potential as a supplementary tool to morphological identification, particularly when dealing with complex species groups or when the insects were in various developmental stages. The integration of these two methods not only enhanced the accuracy of identification but also expanded the scope of pest management strategies. This comprehensive approach ensured a timely and effective response to pest infestations, ultimately safeguarding crop yields and quality. Future research needs to further explore the optimization of DNA barcoding protocols for different pest species as well as the development of user-friendly diagnostic kits for on-site identification, thereby accelerating the adoption of this technology in practical agricultural settings.

Garlic is a geographical indication product of Qi County, Henan Province. It is the pillar industry of agricultural economy and farmer income in Qi County. It is of great significance to the comprehensive implementation of the rural revitalization strategy. In this study, *D. platura* was proved as a dominant catastrophic pest of garlic in Qi County. Due to the change in planting structure, the food web structure of arthropods in the garlic field ecosystem was complicated. Field investigation found that the natural enemy was the leading factor causing the death of young larvae and eggs in the case of pesticide reduction. G. distincta, as a widespread predatory insect in Huang-Huai-Hai region field, was still in the undeveloped area. Our study examined the predatory relationship between insects in the garlic field and *D. platura*, under the influence of landscape patterns and intensive agricultural planting methods, to provide an early foundation for biological control in the garlic field.

Our study revealed that *G. distincta* in the garlic field exhibited a significant predatory preference for the larvae and pupae of *D. antiqua*, which might be closely linked to their feeding habits and prey availability. In garlic field ecosystems, the larvae and pupae of *D. antiqua* constituted the primary food source for *G. distincta*, and the predatory activity of *G. distincta* contributed to reducing pest populations, thereby mitigating damage to garlic crops. Future research should further explore the predatory efficiency and ecological functions of *G. distincta* under varying environmental conditions as well as its potential application in biological pest control. By delving deeper into the biological characteristics and predatory behaviors of *G. distincta*, we may more effectively utilize this natural enemy resource, offering innovative strategies and methods for pest management in garlic fields.

In this study, we found that the third instar of *G. distincta* nymphs and adults raised by garlic root maggots still had good predation ability for *D. platura* larvae and pupae, indicating that the nymph can be used as a suitable insect source for natural enemies. We found that adults of *G. distincta* could make the larvae of *D. platura* die directly. The predation characteristics were better than the parasitic natural enemies and microbial pesticide agents because in using the latter for control in the field, the pests could still be active, continuing to harm the corp for a period of time. In using *G. distincta* as a natural insecticide, it has the characteristic of quick action [14]. Further research is needed to determine the ability to hunt *D. platura* in garlic fields in the future.

In this study, we found that *G. distincta* nymphs and adults showed good predation ability on first to third instar larvae of *D. platura*. The larvae of pests such as *D. platura* were usually harmful in the ground, but mostly on the ground and underground [15]. Both had similar ecological niches in the garlic field. This insect was recognized as a broad-spectrum night predator as early as 1959, could prey upon 10–20 of the larvae of *Prodenia litura* Fabricius in a cotton field [16]. The field experiment by Price et al. showed that each day, two pupae, 37.9 instar larvae, were consumed [17]. This insect could also prey on cotton aphid, oblique night moth larvae and eggs, with a broad spectrum of prey range [18]. Therefore, *G. distincta* could not only serve as the natural enemy of *D. platura* but also prey on other pests in the garlic field. In the future, this kind of insect can be used as a natural enemy resource in the garlic field ecosystem and employed as an effective part of the ecological control of garlic field pests.

Our study found that with the increase in the instar of *D. platura*, the amount of predation gradually decreased, but in the case of satiety, they still searched for prey. After tearing the prey body wall, they only ate a small amount of the body wall content, that is, to look for other prey larvae and continue to fix and bite with the tail clip. In feeding on *D. platura* larvae, as a fertility test, the prey made fierce resistance, young nymphs quickly escaped to avoid prey, fifth instar nymphs and male and female adults could spray the foul smell for defense, then use the tail clip quickly fixed to the prey skin to bite its contents. This indicated that even when encountering individuals larger than the predators, which was similar to most predatory insects [19,20,21], the chemical defense was the main defense skill of the *G. distincta*. In the natural world, releasing the defense gas indicated that the ability to adapt to the environment and predation were strong.

The forcing experiment in this study showed that *G. distincta* could prey the intact pupal body of *D. platura*, and the feeding selection experiment also found that *G. distincta* preferred the active prey. Under the forced situation, *Labidura riparia* did not feed on the pupae of armyworm and cotton bollworm [22,23]. Our experiments indicated that the colonial *G. distincta* could feed on pupae. Similar results were also found in *Chelisoches morio* (Fabricius), which could also prey on *Brontispa longissima* (Gestro) [24,25]. The pupae of *D. platura* were smaller than that of armyworm, which was beneficial to biting the breeding fertilizer. Further research is necessary to determine this in the garlic field.

In the central plains region of China, where there is a large population and limited land, the multifunctional use of land and agricultural intensification simultaneously affect the natural enemy community and its related ecosystem services. The biodiversity of Lepidoptera in garlic fields and their role in food webs remained poorly understood. This study provided preliminary insights for further ecological research on their functional roles. Certain predatory individuals among Dermaptera species serve as crucial natural enemies of agricultural pests. The diversity of earwigs in garlic fields and their role in the food web remained unclear. This study provided a preliminary reference for the in-depth study of its ecological service function. Some predators are important natural enemies of pests in agricultural production, but there are some omnivorous and herbivorous earwigs who also eat seeds, seedlings, flowers, or fruits of crops and become agricultural or storage pests [26,27,28].

In this study, we only determined the pest control ability of *G. distincta* against *D. platura*, and it is necessary to test its pest control ability against other non-premier garlic maggots and apply it in the field in the future.

## Figures and Tables

**Table 1:** COI gene information of root maggot samples and earwigs collected from garlic field.

Root Maggots and Earwig Samples Collected from the Same Garlic Field	GenBank/BOLD Number	Gene Fragment Length/bp	Name of Insect Species
1	NC_085745.1	681	*D. platura*
2	NC_061662.1	688	*B. odoriphaga*
3	NC_028226.1	684	*D. antiqua*
4	NC_034805.1	680	*M. angustifrons*
5	NW_023995419.1	686	*L. sericata*
6	LC767867.1	658	*G. marginalis*

**Table 2:** Daily predation ability of *Gonolabis distincta* on larvae, pupae, and adults of *Delia platura*.

	*D. platura*			
*G. distincta*	1st Instar Larvae	2nd Instar Larvae	3rd Instar Larvae	Pupae
1st instar nymph	(1.85 ± 0.13) Da	(1.75 ± 0.20) Ca	(1.0 ± 0.16) Db	(0.95 ± 0.09) Db
2nd instar nymph	(2.05 ± 0.15) Da	(1.95 ± 0.15) Ca	(1.35 ± 0.17) Db	(1.30 ± 0.18) Dd
3rd instar nymph	(2.45 ± 0.18) Da	(2.15 ± 0.24) Ca	(1.60 ± 0.13) Db	(1.45 ± 0.18) Db
4th instar nymph	(24.00 ± 0.57) Ca	(21.05 ± 0.43) Bb	(20.65 ± 0.31) Cc	(17.30 ± 0.51) Cc
5th instar nymph	(36.00 ± 0.57) Ba	(28.85 ± 0.29) Bb	(24.80 ± 0.21) Cc	(14.25 ± 0.55) Cd
Female adults	(71.25 ± 0.66) Aa	(67.60 ± 0.38) Aa	(57.70 ± 0.54) Ab	(31.40 ± 0.48) ABd
Male adults	(63.85 ± 0.41) ABa	(53.45 ± 0.26) Ab	(42.75 ± 0.41) ABc	(41.40 ± 0.18) Ac

Different uppercase letters in the same column and different lowercase letters in the same row indicated significant differences in the predatory numbers of *G. distincta* in the same developmental stage preying on *D. platura* in different developmental stages and significant differences in the predatory numbers of *G. distincta* in different developmental stages feeding on *D. platura* in the same developmental stages, respectively (*p* < 0.05, Duncan’s multiple range test).

**Table 3:** Predation preference of third instar larvae, female and male adults of *Gonolabis distincta* Nishikawa for larvae and pupae of *Delia platura* Meigen per day.

*G. distincta*	*D. platura*							
	1st Instar Larvae		2nd Instar Larvae		3rd Instar Larvae		Pupae	
5th instar nymph	PN	Ci	PN	Ci	PN	Ci	PN	Ci
	(27.75 ± 0.27) Aa	0.097 Bb	(22.75 ± 0.48) Ab	0.14 Aa	(22.50 ± 0.3) Bb	0.13 ABa	(18.40 ± 0.2) Cc	−0.11 Bb
Female adults	(28.80 ± 2.24) Aa	0.24 Ba	(28.40 ± 0.75) Aa	0.37 Aa	(27.45 ± 2.14) Aa	0.35 Ab	(21.35 ± 1.14) Ab	0.09 Ca
Male adults	(28.6 ± 0.34) Aa	0.06 Cb	(27.7 ± 0.15) Aa	0.38 Ab	(27.3 ± 0.16) Aa	0.18 Bb	(19.9 ± 0.37) Bb	−0.14 Da

The data in the table are presented as mean ± SE. Different capital letters follow the same row, indicating the significant difference among predatory number (preference index) of same developmental *G. distincta* preying on different development stages of *D. platura* (*p* < 0.05), and different lowercase letters follow the same column, indicating the significant difference among predatory number (preference index) of different developmental *G. distincta* feeding on same development stages of *D. platura* (*p* < 0.05) (Duncan’s multiple comparisons). PN: Daily predatory number; Ci, preference index.

## Data Availability

The original contributions presented in this study are included in the article. Further inquiries can be directed to the corresponding authors.
